# Genetic, clinical and biochemical characterization of a large cohort of patients with hyaline fibromatosis syndrome

**DOI:** 10.1186/s13023-019-1183-5

**Published:** 2019-08-27

**Authors:** Claudia Cozma, Marina Hovakimyan, Marius-Ionuț Iurașcu, Nawal Makhseed, Laila A. Selim, Amal M. Alhashem, Tawfeg Ben-Omran, Iman G. Mahmoud, Nihal M. Al Menabawy, Mariam Al-Mureikhi, Magi Martin, Laura Demuth, Zafer Yüksel, Christian Beetz, Peter Bauer, Arndt Rolfs

**Affiliations:** 1 Centogene AG, Am Strande 7, 18057 Rostock, Germany; 2 Department of Pediatrics, Jahra Hospital, Ministry of Health, Jahra City, Kuwait; 3 Division of Neurology and Metabolism, Kasr Al Ainy School of Medicine, Cairo University Children Hospital, Cairo, Egypt; 4 Prince Sultan Military Medical City, Pediatrics, Riyadh, Saudi Arabia; 5 Alfaisal University, Riyadh, Saudi Arabia; 6 Division of Clinical and Metabolic Genetics, Department of Pediatrics, Hamad Medical Corporation, Doha, Qatar; 7 Rostock Medical University, Rostock, Germany

**Keywords:** *ANTXR2*, Biomarker, Farber disease, Genotype-phenotype correlation, Hyaline fibromatosis syndrome

## Abstract

**Background:**

Hyaline fibromatosis syndrome (HFS) is a rare clinical condition in which bi-allelic variants in *ANTXR2* are associated with extracellular hyaline deposits. It manifests as multiple skin nodules, patchy hyperpigmentation, joint contractures and severe pain with movement. HFS shows some clinical overlap to Farber disease (FD), a recessive lysosomal storage disorder.

**Results:**

We here present the largest cohort of independent, genetically confirmed HFS cases reported to date: in 19 unrelated index patients, we identified ten distinct homozygous *ANTXR2* mutations, three of which are novel frame-shift variants. The associated clinical data are consistent with the previous hypothesis of non-truncating variants in the terminal exons 13–17 to confer rather mild phenotypes. The novel observation of gender-dependent disease manifestation in our cohort received support from a meta-analysis of all previously published cases. Untargeted blood-based metabolomics revealed patient samples to be biochemically distinct from control samples. Numerous potential HFS biomarker metabolites could thus be identified. We also found metabolomics profiles of HFS patients to highly overlap with those from FD patients.

**Conclusions:**

Our study extends the mutational spectrum for HFS, suggests gender-dependency of manifestation, and provides pilot metabolomics data for biomarker identification and a better pathomechanistic understanding of the disorder.

**Electronic supplementary material:**

The online version of this article (10.1186/s13023-019-1183-5) contains supplementary material, which is available to authorized users.

## Background

Hyaline fibromatosis syndrome (HFS, MIM #22860) is characterized by the accumulation of clear (hyaline) substance in body tissues. Such noncancerous masses may grow under the skin and the gums resulting in bumps/nodules and gingival hypertrophy, respectively. Joint stiffness and deformities are frequent, and the skin covering the joints is often hyperpigmented. Villous atrophy and intestinal lymphangiectasia result in severe diarrhea and cachexia. Patients may come to clinical attention from birth to late childhood. The most common initial symptoms include extreme pain at minimal handling and progressive joint contractures [1]. An early onset, more severe, and usually fatal form termed infantile systemic hyalinosis (ISH) had long been differentiated from a later onset and less severe form termed juvenile hyaline fibromatosis (JHF) [2]. The finding of a shared genetic background, however, eventually resulted in the suggestion to use the umbrella term HFS along with a three-partite clinical grading scheme (mild vs. moderate vs. severe) [3]. A refinement to four severity grades was proposed more recently [4].

HFS is a recessive, genetically homogeneous disorder; it is caused by bi-allelic variants in *ANTXR2* [5] [6]. Approximately 100 genetically confirmed patients which carry a total of 46 distinct HFS-associated variants have been published to date [The Human Gene Mutation Database at http://www.hgmd.cf.ac.uk]. Most frequent are missense alterations (*n* = 19), but clearly inactivating alleles (3 x nonsense, 9 x splice site, 13 x frameshift, 2 x large deletions) collectively predominate. With the exception of a mutational hotspot at c.1072_1076, pathogenic variants are more or less equally distributed over the coding sequence [7].

One of the two initial papers on *ANTXR2* mutations in HFS suggested that non-truncating variants which affect the protein’s C-terminus (encoded by exons 13–17) are associated with comparatively late disease onset and a rather benign disease course [5]. Two subsequent meta-analyses found additional support for this hypothesis [7] [8]. The overall rarity of HFS, however, has hampered the search for further genotype-phenotype correlations.

The *ANTXR2* gene had initially been designated *CMG2* (capillary morphogenesis gene 2), and this was based on the observation of elevated expression in vein endothelial cells induced to undergo capillary formation [9]. The subsequent finding of the encoded protein to serve as a receptor for the bacterial anthrax toxin resulted in the renaming to *ANTXR2* (anthrax toxin receptor 2) [10]. The encoded ANTXR2 protein interacts with several components of the extracellular matrix [9]. It has further been suggested to serve as a collagen receptor that mediates collagen transport to lysosomes [11]. Impaired degradative processes may therefore contribute to the accumulation of hyaline material in HFS patients. The complete spectra of the physiological and pathological roles of ANTXR2, however, remain to be defined.

HFS shows considerable clinical overlap to Farber disease (FD), an autosomal recessive, infantile onset lysosomal storage disorder [12]. FD is, in fact, the main differential diagnosis for HFS, with painful and swollen joints as well as periarticular and subcutaneous nodules being the most prominent shared symptoms [1, 13]. FD is caused by mutations in *ASAH1*, the gene encoding acid ceramidase [14]. This lysosomal hydrolase catalyzes the breakdown of ceramides into sphingosines and fatty acids [15]. Applying a targeted metabolomics approach, we recently identified ceramide C26:0 as a highly sensitive, blood-based biomarker for FD [16]. Conceptually similar studies in HFS are currently lacking.

The present study is based on a large cohort of patients who presented with symptoms from the HFS-FD clinical spectrum. We set out to genetically characterize this cohort, and to utilize it in the search for novel genotype-phenotype correlations. We also applied an untargeted metabolomics approach in order to gain preliminary biochemical insight into HFS.

## Results

### Nineteen unrelated index cases are homozygous for known or novel variants in *ANTXR2*

Sanger sequencing of the *ANTXR2* gene, as requested for nine patients that had received a clinical diagnosis of HFS, readily identified a single homozygous variant in eight cases. In the remaining sample, no sequence aberrations were found, but attempts to amplify exons 15 and 16 repeatedly failed. This finding suggests presence of a large homozygous deletion which encompasses *ANTXR2* exons 15 and 16. Five additional patients, for which extended Sanger sequencing was initiated following negative *ASAH1* screening, were also found positive for homozygous *ANTXR2* variants. Finally, in five patients for which WES had been requested and which showed a suggestive phenotype, homozygous *ANTXR2* variants were identified. For four of the 19 index patients, material from two to six unaffected family members was available. These were all found to not carry the familial variant or to be heterozygous carriers (true for all available parental samples). All 19 patients thus received a genetic diagnosis of *ANTXR2*-related HFS (Table 1).

**Table 1:** Genetic and clinical findings for the 19 unrelated patients analyzed by the present study

IndividuaI’s ID	initial diagnostic request	gender	parental consan-guinity	age at referral [months]	subcuta-neous nodule	skin abnormalities	skeletal or muscle abnormalities	pain	joint involvement	coarse facies	visceral involvement	*ANTXR2* variants identified (all in homozygous state)
1013011	*ANTXR2*	male	n.r.	8	+	+	+		+			c.1074delT
1019835	*ANTXR2*	female	n.r.	248	+		+	+			+	c.1180-?_1428 +?del
1030796	*ANTXR2*	male	n.r.	12	n.r.							c.1074delT
1034217	*ANTXR2*	female	n.r.	13	n.r.							c.1073dupC
1034651	*ANTXR2*	male	yes	6	n.r.							c.134 T > C
1062973	*ANTXR2*	male	yes	8	n.r.							c.1074delT
1083931	WES	female	yes	22			+		+	+		c.134 T > C
1111097	*ANTXR2*	male	n.r.	4				+	+			c.1074delT
1124610	*ANTXR2*	male	yes	1	+	+		+	+			c.134 T > C
1160661	*ANTXR2*	female	yes	65	+	+	+		+		+	c.1294C > T
1167266	WES	female	yes	16	+		+		+			c.652 T > C
1175869	WES	male	yes	3		+	+	+			+	c.720delT
1205436	ASAH1	male	yes	5		+	+	+	+	+	+	c.720delT
1206223	ASAH1	male	n.r.	18	+		+	+	+			c.994delC
1206224	WES	male	n.r.	17	+		+	+	+	+		c.1073delC
1218829	ASAH1	male	yes	10	+	+	+		+	+		c.1074delT
1219558	ASAH1	female	yes	15	+		+		+			c.1074delT
1223185	WES	male	n.r.	9			+		+	+	+	c.134 T > C
1234238	ASAH1	male	n.r.	8	+	+	+	+				c.51delC

*n.r.* not reported

**Fig. 1:**
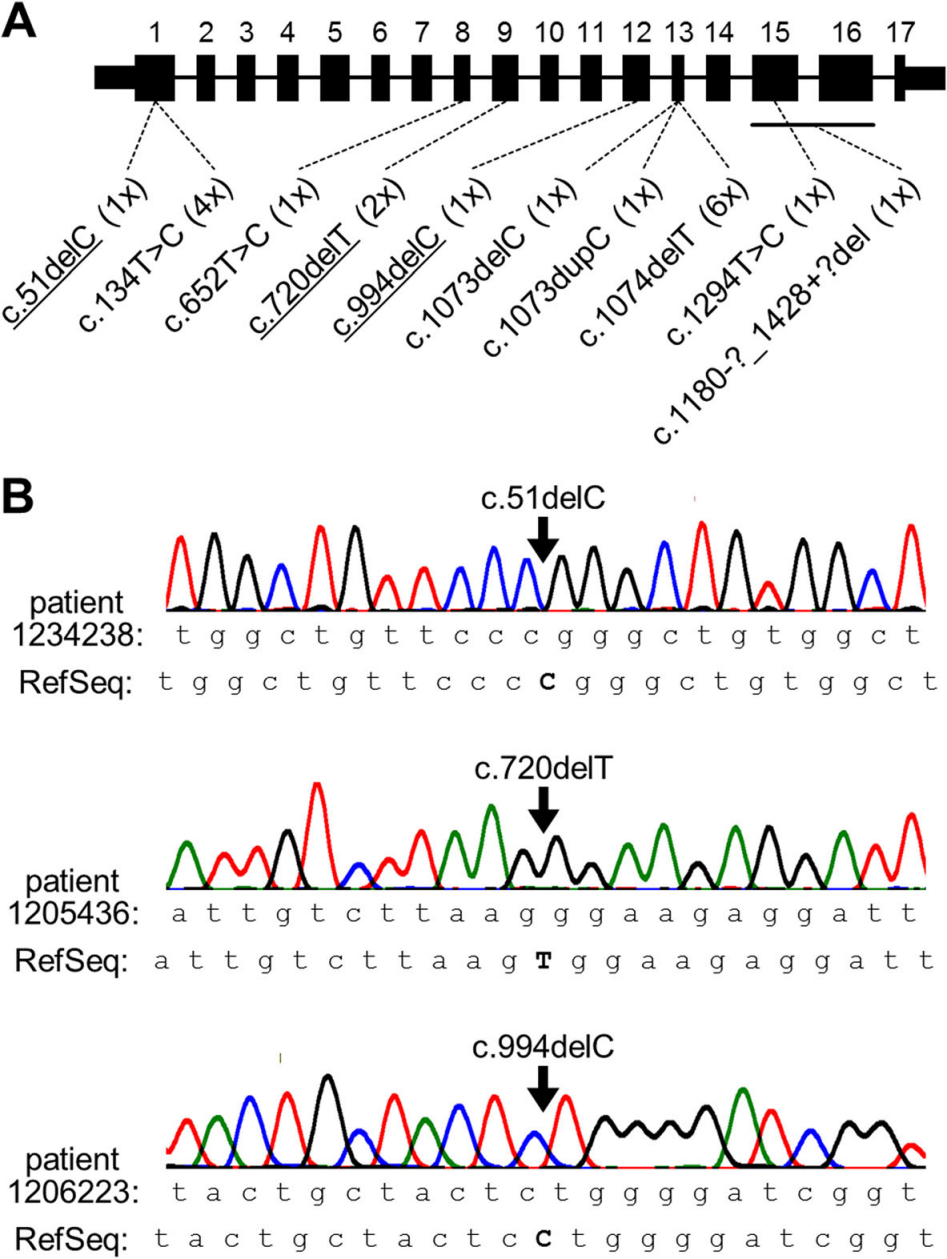
Results of *ANTXR2* mutation screening in 19 unrelated HFS patients. (**a**) Scheme of the 17-exon *ANTXR2* gene (coding parts of exons to scale). The exonic localisation as well as the number of independent observations (in parentheses) of pathogenic homozygous variant are indicated below the scheme. Novel variants are underlined. (**b**) Exemplary Sanger sequencing traces for patients that harbor one of the three novel variants each. RefSeq, reference sequence

In total, there were 10 distinct variants, seven of which have been described previously (Fig. 1a, b; Table 2). By far the most frequently observed variant was c.1074delT (identified 6x), followed by c.134 T > C (4x) and c.720delT (2x). Three distinct variants (c.1073dupC, c.1073delC and c.1074delT) affected a specific dinucleotide in exon 13, whereas the other variants appeared more or less evenly distributed over the coding sequence (Fig. 1a). Six of the ten variants were frameshift variants, two were missense, and one each was a nonsense variant and a large in-frame deletion, respectively (Table 2).

**Table 2:** Novelty and geographic origin of *ANTXR2* variants identified by the present study

Variant	Predicted consequence	Times observed by present study	Novel?	Patient origin in present study	Patient origin in previous studies^†^
3c.51delC	p.L19Cfs*56	1	yes	1 x Africa	–
c.134 T > C	p.L45P	4	–	4 x Middle East	Middle East,
c.652 T > C	p.C218R	1	–	1 x Middle East	Asia
c.720delT	p.S240Rfs*20	2	yes	2 x Middle East	–
c.994delC	p.L332Wfs*5	1	yes	1 x Africa	–
c.1073delC	p.P358Lfs*51	1	–	1 x Africa	Asia
c.1073dupC	p.A359Cfs*13	1	–	1 x Middle East	Middle East, Japan, North America, Latin America, Asia, Europe, unreported
c.1074delT	p.A359Hfs*50	6	–	5 x Middle East, 1 x Latin America	Middle East, Asia, Africa, Latin America, unreported
c.1294C > T	p.R432*	1	–	1 x Asia	Latin America, Asia, unreported
c.1180-?_1428 +?del	p.V394_490del	1	–	1 x Middle East	Latin America

^†^for references see Additional file 3: Table S3

### Clinical presentation of *ANTXR2*-related HFS may be gender dependent

**Fig. 2:**
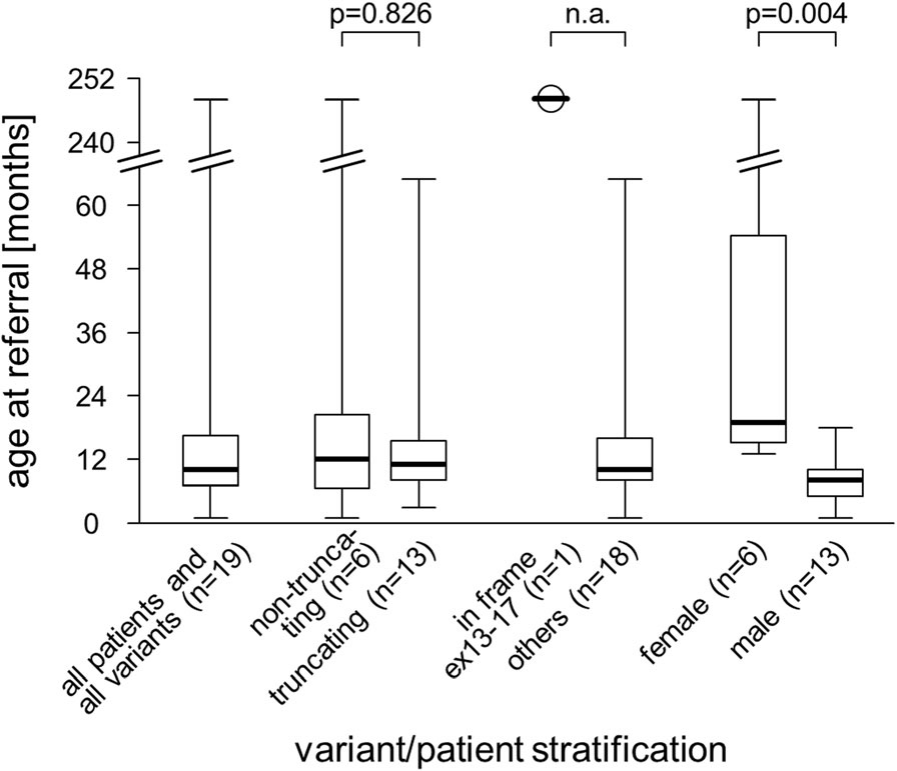
Potential clinical correlations. Age at referral for genetic workup is not associated with variant type, but may be influenced by variant localization, and correlates with gender (*p*-values according to the two-sided Mann-Whitney U-Test; n.a., not applicable)

Clinical information had been provided for 15 of the 19 patients; Table 1 summarizes these findings. The primary phenotypic observations were available as only rudimentary notions for some patients, but in much greater detail for others. This fact precluded a well-founded search for genotype-phenotype correlations. In order to still enable testing for associations, we focused on age at referral/manifestation. We further stratified patients according to the type of variant (truncating vs. non-truncating) and gender, and considered whether the variant is in-frame and in exons 13–17, or not (compare introduction and see Additional file 1: Table S1). While there was no evidence for an impact of the type of variant, the single in-frame variant localizing to exons 13–17 was associated with the maximum age at referral. Moreover, female patients were found to be significantly older than male patients at referral (Fig. 2). As the latter finding was unexpected and as we also noticed a (non-significant) male predominance in our cohort (13 males vs. 6 females), we analyzed gender of all genetically confirmed HFS patients as reported in the literature (Additional file 2: Table S2). We did not observe male predominance (36 males vs. 36 females), but there was a trend for males to more frequently being diagnosed with ISH rather than with JFH (19 male ISH plus 6 male JFH vs. 14 female ISH plus 13 female JFH; *p* = 0.064, one-sided Fisher’s Exact Test).

### Metabolomic profiles from HFS patients are inherently different from those of controls

**Fig. 3:**
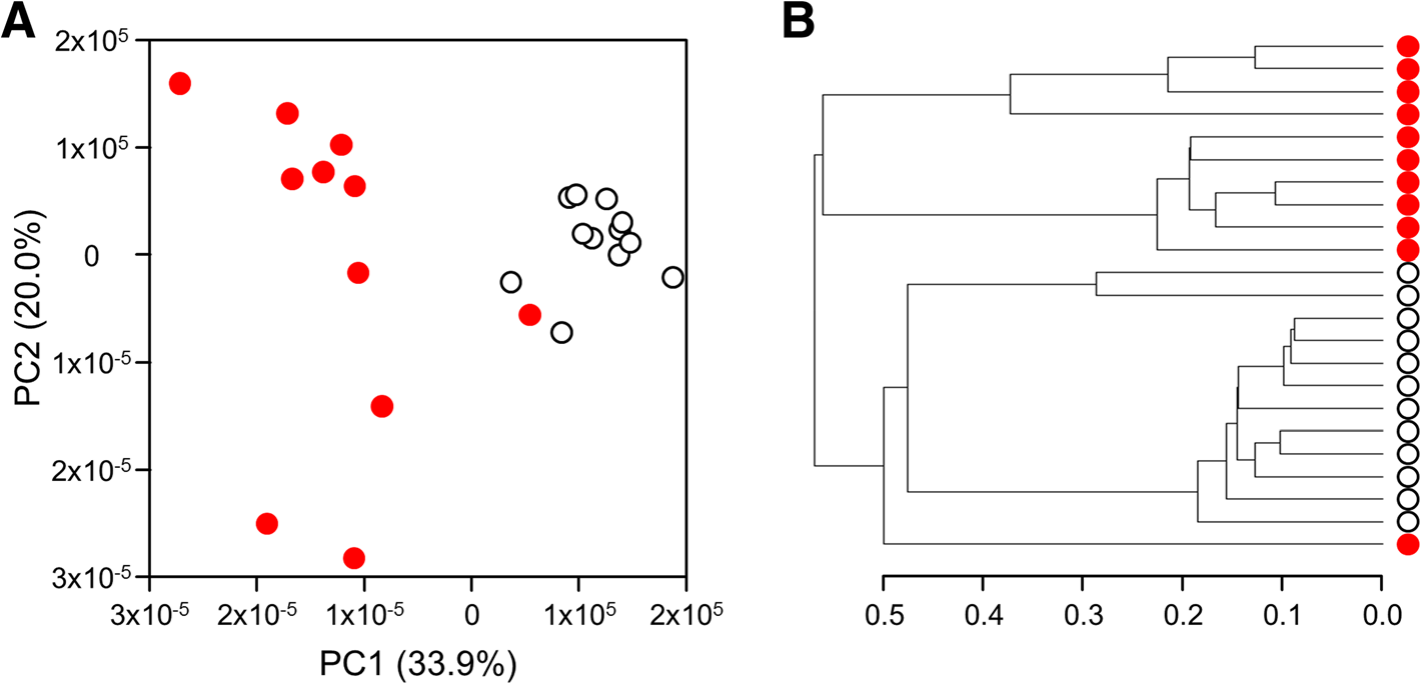
Unsupervised analysis of all 4978 compounds that met our detection criteria as regards quality and quantity upon comparative analysis of samples from HFS (in red) and control samples (in white). (**a**) Principal component analysis separates the majority of HFS patient samples from controls samples, and this is largely based on principal component 1. (**b**) Hierarchical clustering confirms that control samples are biochemically distinct from patient samples

Metabolomic profiling of samples from 11 HFS patients and 12 controls identified a total of 4978 compounds that met our detection criteria as regards quality and quantity. We first analyzed these data in an unsupervised manner. Principal component analysis (PCA) completely separated both types of samples, and this was mainly due to principal component 1 which explains 33.9% of the overall variability (Fig. 3a). Similarly, hierarchical clustering showed that most HFS patient samples are closer related to each other than to any of the control samples (Fig. 3b). These initial observations indicated that the metabolomics data are highly structured, and that this structure is largely dictated by clinical status.

### Numerous individual compounds flag HFS patient samples with 100% sensitivity

**Fig. 4:**
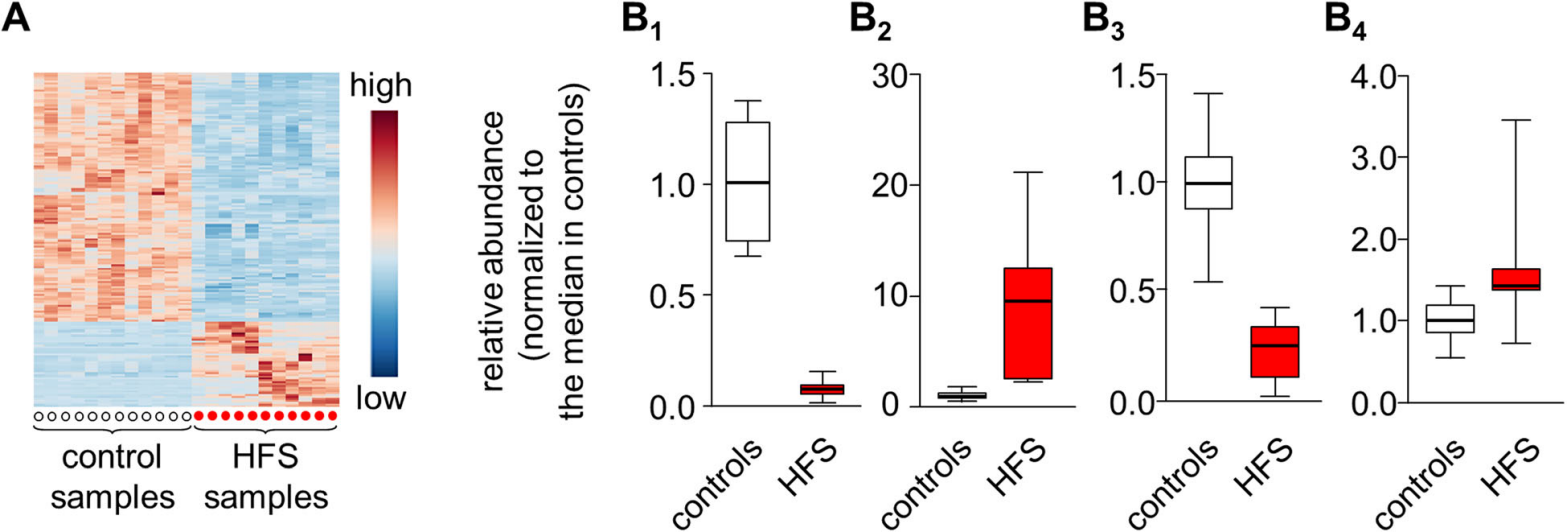
Potential HFS biomarkers. (**a**) Heat map visualizing all 181 compounds for which values in HFS samples do not overlap with values in control samples. Note that the majority of the compounds is decreased in patients. (**b**) Box-plots for selected compounds (control samples in white, HFS patient samples in red). Y-axes indicate fold-changes relative to the median for the control samples. (B_1_) Unknown compound with an m/z ratio of 417.300467 and a charge of 1; (B_2_) Ceramide Cer(d18:0/d22:0); (B_3_) Sphingomyelin SM(d18:1/d16:1). (B_4_) Ceramide C26:0

Given the above finding of a strong overall difference between samples from patients vs. from controls, we next aimed at identifying the very compounds that confer this difference. Considering the biomarker aspect of our study, we not simply searched for compounds with significantly differing values, but for compounds for which the range of values in patients does not overlap with the range in controls. A total of 181 such compounds were identified. 135 of these (75%) were decreased in patients, while 46 (25%) were increased (Fig. 4a).

The identity was unknown for 112 of the 181 candidate compounds, but many of these showed very large differences between patients and controls (e.g. Fig. 4B_1_). For the remaining 69 compounds, mass and charge enabled deducing a likely identity. The corresponding list contained several ceramides (e.g. Fig. 4B_2_) and sphingomyelins (e.g. Fig. 4B_3_), but also di- and triglycerides (not shown). Ceramide 26:0, previously shown by us to be increased in a 10 of 10 FD patients [16], was not amongst the fully sensitive biomarker candidates, but the values in HFS patients were significantly higher than in controls (*p* = 0.01, two-sided Student’s T-test) (Fig. 4B_4_).

### HFS samples and FD samples are biochemically highly similar

**Fig. 5:**
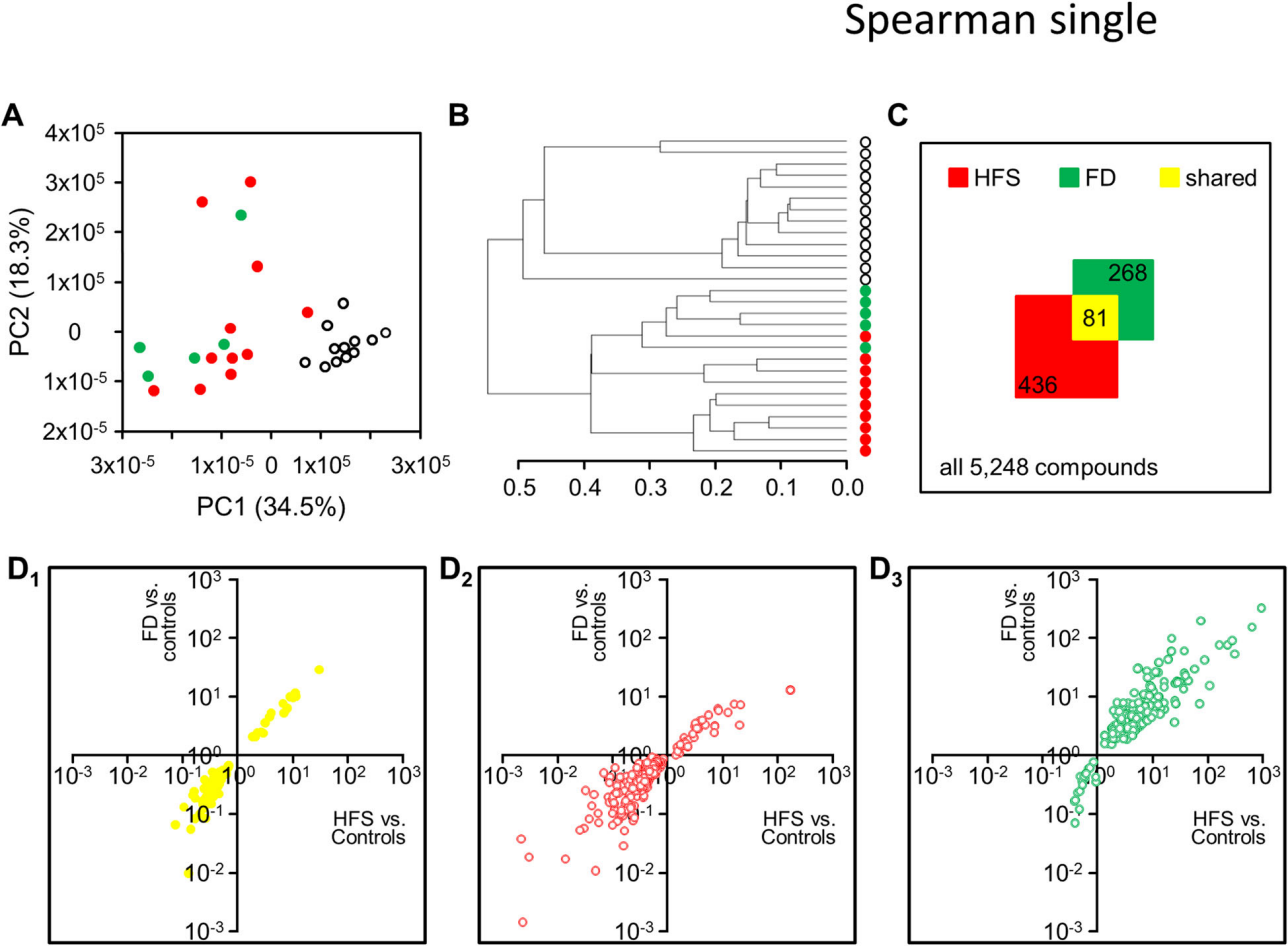
Similarity of metabolic profiles from HFS and FD patients (HFS samples in red; FD samples in green; control samples in white). (**a**) Unsupervised PCA of all 5248 compounds that survived quality and quantity filters separates patient samples from controls samples, and this is largely based on principal component 1 (compare Fig. 3a. **b** Unsupervised hierarchical clustering confirms that patient samples are biochemically distinct from control samples, and additionally suggests that HFS patients and FD patients differ in their overall metabolomic profiles. (**c**) To-scale scheme visualizing all 5248 compounds (large square) in relation to the number of disease-specific compounds as specified. (**d**) Compound-specific fold changes (mean value for disease samples divided by mean value for control samples) for the 81 compounds which differed significantly from controls in both HFS and FD (D_1_), in only HFS (D_2_), or in only FD (D_3_)

Based on the clinical overlap between HFS and FD, and on finding ceramide 26:0 to be increased in both conditions, we aimed at comparing the metabolomics profiles from HFS patients and FD patients. Applying our predefined quality and quantity criteria, 5248 compounds survived filtering. Principal components 1 and 2 in PCA (collectively explaining 52.8% of the overall variability) revealed the samples from both types of patients to collectively define a larger entity which is rather diverse, but clearly distinct from control samples (Fig. 5a); compare (Fig. 3a). Unsupervised hierarchical clustering confirmed this observation, and, in addition, revealed evidence for disease-specificity of the metabolomics profiles (Fig. 5b).

We finally defined all compounds for which values differed significantly from control values in HFS patients and in FD patients. We thereby identified 436 compounds for HFS and 268 compounds for FD. 81 compounds were shared, and this finding is highly significant (*p* = 7 × 10^− 18^, two-sided Fisher’s Exact Test) (Fig. 5c). Moreover, the direction of change was the same in HFS and FD samples for all 81 compounds (Fig. 5D_1_). This was also true for every compound which differed significantly from controls in only HFS samples (Fig. 5D_2_) or in only FD samples (Fig. 5D_3_).

## Discussion

HFS is a very rare disorder. Most previous clinical-genetic studies have therefore been able to present one or a few cases only. With a size of *n* = 19, our cohort significantly increases the number of known independent patients with genetically confirmed HFS from 74 to 93 (compare Additional file 2: Table S2). Together with the pioneering paper by Hanks et al. [5], in which 18 families were described, our study thereby represents the largest genetic report on HFS.

All of our patients were found to be homozygous for pathogenic *ANTXR2* variants, while 21% of previously published cases were compound heterozygous [8]. Considering our cohort to contain many consanguineous families (Table 1), and to generally derive from regions with a high degree of consanguineous marriages (Table 2), the above observation is not surprising. Geographic origin may also serve to explain recurrent identification of certain variants (Table 2). This is probably true for c.1074delC, which was present in six of our patients and has previously been associated with a specific haplotype [17]. There is evidence for further founder variants, but also for *ANTXR2* mutational hotspots [5]. A more detailed investigation of this issue in our cases, however, was beyond the scope of the diagnosis-focused concept of the present study.

Three of the ten variants we observed have not been reported previously (Fig. 1a, Table 2). Our genetic findings thereby increase the number of known pathogenic *ANTXR2* variants to 49 [The Human Gene Mutation Database at http://www.hgmd.cf.ac.uk]. All three novel variants are deletions of single nucleotides in rather 5′-situated or central exons, and are therefore predicted to trigger nonsense-mediated decay [18]. They thus represent bona fide loss-of-function variants, supporting the hypothesis of HFS to be mediated by absence of ANTXR2 or complete functional inactivation [19].

The phenotypes of all patients for which clinical information was available were consistent with the well-known, though wide-ranging spectrum of manifestations of HFS (Table 1) [1]. Pertinent information, together with the comparatively large size of our cohort enabled us to analyze potential clinical correlations. The only corresponding finding from previous studies was that variants which affect the protein’s cytoplasmic tail (encoded by the terminal exons 13–17) and are predicted to not result in mRNA instability are associated with an overall milder disease and a later onset [5, 7, 8]. As there was only one patient with such a variant in our cohort (Additional file 1: Table S1), a formal statistical analysis was not possible. However, the fact that this patient was > 20 years and alive at referral strongly supports a comparatively mild nature of the corresponding in-frame deletion (Fig. 2). We next stratified patients more generally according to the type of variant. This was based on the observation of non-truncating variants to be less detrimental than truncating variants in some genes (e.g. ref. [20]). We did, however, not find evidence for an impact of the type of *ANTXR2* variant on age at onset of HFS (Fig. 2). When finally considering the gender of patients, we noticed considerable male predominance in our cohort, and found our male patients to be significantly younger at referral (Fig. 2). Given the geographic background of our cohort (Table 2), this observation may partially be explained by cultural factors that favor males over females in access to health care [21]. We therefore initiated an exhaustive literature analysis. Though age-related data could not be compiled in a sufficiently uniform manner, there was a trend for females to more often being diagnosed with JFH rather than ISH (Additional file 2: Table S2), which indicates an overall milder manifestation and later onset [3]. Together with the fact that there was no evidence for male predominance amongst previously published cases (Additional file 2: Table S2) [8], this findings argues against a major impact of the above cultural factors. A gender-dependency of the clinical consequences of *ANTXR2* mutations may thus be real. Though very rare, the phenomenon of gender-specific disease manifestation has been reported for other autosomal genes (e.g. refs. [22-24]). Understanding its pathological basis in HFS may eventually result in hitherto unexplored therapeutic options.

As far as we are aware, our biochemical characterization of samples from HFS patients is the first pertinent effort published to date. It was facilitated by both the size of our HFS cohort and the availability of DBS samples. Given the lack of hypotheses about the impact of *ANTXR2* variants on certain blood metabolites, we had chosen an untargeted approach. Unsupervised analyses revealed that patient metabolomes are inherently different from control metabolomes (Fig. 3). Part of this overall difference, though, may be related to the lack of age- and gender-matching in our study. Indeed, metabolomics profiles have been shown to both change over time and differ between genders [25-27]. Ranges of values in corresponding studies, however, highly overlap and mean fold-changes rarely exceed 3, and this is in stark contrast to what is observed for our set of data (Fig. 4). Another factor that may conceptually affect a comparison between patient and control metabolomes is medication [28]. For HFS, however, nonsteroidal anti-inflammatory drugs and opiates represent the only potentially shared drugs [1], and these are not expected to have major influences. We thus considered the majority of the metabolic differences to be truly related to clinical/mutational status.

Our attempt to define potential metabolomics biomarkers for HFS resulted in a list of 181 candidate compounds that are associated with maximum discriminatory power (i.e. 100% sensitivity) for our patient vs. control cohorts. Though the inclusion of larger numbers of samples can be expected to result in a reduced list and in a drop in sensitivity, this observation of our pilot study is highly promising. In addition to the primarily diagnostic aspect addressed here, some of the compounds may eventually turn out to be of further relevance, e.g. for monitoring disease progression and drug response, for a stratification of patients, and/or for a better understanding of the underlying pathology [29].

A phenotypic overlap of HFS and FD has long been recognized [1], and our clinical-genetic findings (Table 1) re-inforce the notion that a primary clinical diagnosis of FD may need to be corrected to HFS upon genetic work-up (e.g. ref. [30]). With FD resulting from an enzyme deficiency [31] and HFS being due to inactivation of what is likely an extracellular collagen receptor [11], additional analogies at the level of pathobiochemistry would not necessarily be expected. Our comparative analysis still suggested that the phenotypic similarity of HFS and FD extends to the blood metabolomics signatures (Fig. 5). Future studies will be needed to see whether this observation is due to a sharing of the primary cellular defect(s) and, thus, to common potential targets for therapeutic interventions.

## Conclusions

The present paper reports on a comparatively large number of previously unreported patients with HFS, and thereby significantly extends the mutational and clinical spectra associated with this disease. A previously suggested genotype-phenotype correlation received further support, and gender-dependency of manifestation is suggested as a previously unrecognized phenomenon. The additional metabolomics findings represent a promising basis for the development of HFS-specific biomarkers, and for understanding the pathophysiology of the disease. Our study thereby provides valuable novel insights into this very rare genetic condition.

## Methods

### Patients

The present study enrolled 19 unrelated patients referred for genetic diagnostic workup of presumably congenital phenotypes to (Rostock, Germany) Centogene AG. Nine of them had received an expert clinical diagnosis of HFS, and targeted *ANTXR2* sequencing was requested. For five patients, the initial genetic diagnostic request had been targeted *ASAH1* sequencing based on a clinical suspicion of FD. Whole exome sequencing (WES) was requested for the remaining five patients (Table 1). For a subset of the above index cases, samples from unaffected family members were provided, too. The most frequent region of origin was the Middle East, followed by Africa, Latin American and Asia (compare Table 2). For metabolomic profiling, eleven HFS patients five patients with genetically confirmed FD and 12 healthy controls were included [16].

### DNA preparation

Samples were provided as ready-to-use DNA, EDTA blood, or as dried blood spots (DBSs) on filter cards (CentoCard®, Centogene AG). Extraction from the blood-based samples utilized QIAsymphony instruments in combination with reagents and kits as recommended by the manufacturer (Qiagen, Hilden, Germany).

### *ANTXR2* variant screening

The coding sequence of *ANTXR2* (NM_058172.5; NP_477520.2) including at least 50 bp of adjacent untranslated regions or intronic sequences was amplified exon-wise from genomic DNA (primers available upon request). PCR-products were extracted from agarose gels, purified according to standard procedures, and sequenced from both sides on a 3730*xl* sequencer (Thermo Fisher Scientific, Waltham, MA).

### Metabolomic profiling

Three DBS punches of 3.2 mm in diameter were prepared from filtercards using a DBS puncher (Perkin Elmer LAS, Germany), and collected into 2.2 ml round bottom tubes (Eppendorf, Germany). Extraction was performed by adding 50 μL extraction solution (DMSO:H_2_O, 1:1) and 100 μL internal standard solution (lyso-Gb2, Matreya LLC, USA, 200 ng/mL in ethanol). After a brief vortex-mixing, the tubes were shaken (700 rpm) at 37 °C for 30 min and then sonicated at maximum power for 1 min. All liquid was subsequently transferred to an AcroPrep Filter Plate with PTFE membrane (PALL, Germany) placed on a 96 well V-shape bottom plate (VWR, Germany). To remove solid particles, samples were filtrated by centrifugation at 3.500 rpm for 5 min.

Mass spectrometric (MS) analysis was performed on a Waters Acquity i class UPLC (Waters, UK) coupled with a Vion IMS-QTof mass spectrometer (Waters, UK). Chromatographic run was performed on a Kinetex EVO C18 column (Phenomenex, Germany) with a gradient from 0 to 100% organic solvent (50 mM formic acid in acetonitrile:methanol, 1:1, v:v). Mass spectrometric acquisition was made using the following parameters: analyzer mode - sensitivity, MS mode - High definition MSE, capillary voltage - 1.2 kV, source temperature - 150 °C, desolvation temperature - 600 °C, desolvation gas - 1000 L/h, cone gag - 50 L/h, low collision energy - 6 eV, high collision energy ramp: 20–40 eV, scan mass: 50–1000 m/z, scan time - 0.5 s.

10 μL samples were injected and an HDMSE analysis method was used. The acquisition was done using the Unifi software (Waters, UK) and the results exported as a Unifi export file (.uep). The results were imported in the Progenesis QI software (Nonlinear Dynamics, UK) for statistical interpretation. From the list of identified compounds, only those with significant difference between the groups were selected for further use. Targeted mass spectrometry-based screening for the levels of ceramide C26:0 in DBSs was performed as described in detail previously [16].

### Normalization, filtering and analysis of metabolomics data

Raw abundances as detected by untargeted MS were normalized using default settings in Progenesis. Compounds with a charge of > 5 and a mass-to-charge ratio (m/z) < 179 were removed (quality filters). For comparative analyses, only compounds with a median normalized abundance of > 100 counts relative to the reference compound in at least one of the groups under consideration were retained (quantity filter).

Normalized and filtered abundances were transformed into CSV files, and uploaded into the ‘Statistical Analysis’ tool-box of MetaboAnalyst 4.0 at http://www.metaboanalyst.ca [32]. Principal component analysis was performed using default settings. Dendrograms were derived using distance measure ‘Spearman’ and clustering algorithm ‘Single’. Distributions and ranges for values were visualized by generating heat-maps with enforced sample grouping.

## Additional files

Additional file 1: **Table S1.** Data that were used to test for a correlation with age at referral (compare Fig. 2). (DOCX 13 kb)

Additional file 2: **Table S2.** Summary of previously published, clinical-genetic studies on ANTXR2-related HFS. (DOCX 22 kb)

Additional file 3: **Table S3.** References for previous reports on ANTXR2 variants that were also identified by the present study (compare Table 2). (DOCX 16 kb)

## Data Availability

The datasets generated and/or analyzed during the current study are available from the corresponding author on reasonable request.
