# Supplementary material for: Genetic, clinical and biochemical characterization of a large cohort of patients with hyaline fibromatosis syndrome
Source: Orphanet J Rare Dis. 2019 Aug 27;14:209. doi: 10.1186/s13023-019-1183-5 (PMC6712857; doi:10.1186/s13023-019-1183-5)
Supplement: Supplementary file 1 — Table S1. Data that were used to test for a correlation with age at referral (compare Fig. 2). (DOCX 13 kb) [file 13023_2019_1183_MOESM1_ESM.docx]

**Supplementary Table 1.** Data that were used to test for a correlation with age at referral (compare Figure 2).

| **individuaI’s ID** | **age at referral**  **[months]** | **type of**  ***ANTXR2* variant** | **in-frame and**  **in exons 13-17** | **gender** |
| --- | --- | --- | --- | --- |
| 1013011 | 8 | truncating | no | male |
| 1019835 | 248 | non-truncating | yes | female |
| 1030796 | 12 | truncating | no | male |
| 1034217 | 13 | truncating | no | female |
| 1034651 | 6 | non-truncating | no | male |
| 1062973 | 8 | truncating | no | male |
| 1083931 | 22 | non-truncating | no | female |
| 1111097 | 4 | truncating | no | male |
| 1124610 | 1 | non-truncating | no | male |
| 1160661 | 65 | truncating | no | female |
| 1167266 | 16 | non-truncating | no | female |
| 1175869 | 3 | truncating | no | male |
| 1205436 | 5 | truncating | no | male |
| 1206223 | 18 | truncating | no | male |
| 1206224 | 17 | truncating | no | male |
| 1218829 | 10 | truncating | no | male |
| 1219558 | 15 | truncating | no | female |
| 1223185 | 9 | non-truncating | no | male |
| 1234238 | 8 | truncating | no | male |
|  | **median=10 months**  **(range 1 to 248 months)** | **13 truncating**  **vs.**  **6 non-truncating** | **18 x no**  **vs.**  **1 x yes** | **13 males**  **vs.**  **6 females** |
