# Supplementary material for: Genetic, clinical and biochemical characterization of a large cohort of patients with hyaline fibromatosis syndrome
Source: Orphanet J Rare Dis. 2019 Aug 27;14:209. doi: 10.1186/s13023-019-1183-5 (PMC6712857; doi:10.1186/s13023-019-1183-5)
Supplement: Supplementary file 2 — Table S2. Summary of previously published, clinical-genetic studies on ANTXR2-related HFS. (DOCX 22 kb) [file 13023_2019_1183_MOESM2_ESM.docx]

**Supplementary Table 2.** Summary of previously published, clinical-genetic studies on *ANTXR2*-related HFS.

| **study** | **families total** | **patients total** | **patients gender provided** | **females total** | **males total** | **female ISH** | **female JHF** | **male ISH** | **male JHF** |
| --- | --- | --- | --- | --- | --- | --- | --- | --- | --- |
| Aggarwal_2016_J Orthop Case Rep | 1 | 2 | 2 | 0 | 2 |  |  | 2 |  |
| Al Sinani_2013 Oman Med J | 1 | 1 | 1 | 1 | 0 | 1 |  |  |  |
| Antaya_2007_Am J Dermatol | 1 | 1 | 1 | 1 | 0 |  | 1 |  |  |
| Casas-Alba_2018_Hum Mut | 1 | 1 | 1 | 1 | 0 | *no stratification of patient as ISH vs. JHF* | | | |
| Denadai_2012_Am J Med Genet A | 4 | 5 | 5 | 2 | 3 | 2 |  | 2 | 1 |
| Deuquet_2011_EMBO MOL Med | 4 | 4 | 4 | 1 | 3 |  | 1 | 2 | 1 |
| Dowling_2003_AJHG | 4(3) ^a^ | 5(3) ^a^ | 5(3) ^a^ | 4(3) ^a^ | 1(0) ^a^ | 2 (1) ^a^ | 2 | 1 (0)^a^ |  |
| El-Kamah_2010_Br J Dermatol | 2 | 6 | 4 | 1 | 3 | 1 |  | 1 | 2 |
| Fong_2012_Cin Exp Dermatol | 1 | 1 | 1 | 1 | 0 | 1 |  |  |  |
| Haidar_2017_BMC Genet | 1 | 5 | 5 | 0 | 5 | *no stratification of patients as ISH vs. JHF* | | | |
| Hakki_2005_J Clin Peridontol | 1 | 2 | 2 | 1 | 1 |  | 1 |  | 1 |
| Hanks_2003_AJHG | 18 | 30 | 4 | 2 | 2 | 1 | 1 | 1 | 1 |
| Hatamochi_2007_Br J Dermatol | 1 | 1 | 1 | 1 | 0 |  | 1 |  |  |
| Huang_2007_Br J Dermatol | 1 | 1 | 1 | 0 | 1 |  |  | 1 |  |
| Jaoud_2014_J Med Case Rep | 1 | 1 | 1 | 0 | 1 | *no stratification of patients a ISH vs. JHF* | | | |
| Koonuru_2015_Intractable & Rare Dis Res | 1(0)^b^ | 2(0)^b^ | 2(0)^b^ | 0 | 2(0)^b^ |  |  | 2(0)^b^ |  |
| Krasuska-Slawinka_2015_J Oral Maxillofac Surg | 1 | 1 | 1 | 0 | 1 |  |  | 1 |  |
| Lee_2005_Clin Exp Dermatol | 1 | 1 | 1 | 1 | 0 | 1 |  |  |  |
| Lindvall_2008_J Am Acad Dermatol | 1 | 1 | 1 | 1 | 0 | 1 |  |  |  |
| Mallet_2010_Ann Dermatol Venereol | 1 | 1 | 1 | 1 | 0 |  | 1 |  |  |
| Mohamed_2014_Pediatr Neonatol | 1 | 1 | 1 | 1 | 0 | 1 |  |  |  |
| Narayanan_2016_Ind J Pediatr | 1 | 1 | 1 | 0 | 1 |  |  | 1 |  |
| Olczak-Kowalczyk_2011_Eur Arch Pediatr Dent | 1(0)^c^ | 1(0)^c^ | 1(0)^c^ | 0 | 1(0)^c^ |  |  | 1(0)^c^ |  |
| Pena_2018_Genet Med | 1 | 1 | 1 | 1 | 0 | 1 |  |  |  |
| Pirgon_2007_J Pediatr Endocrinol Metab | 1 | 1 | 1 | 1 | 0 | 1 |  |  |  |
| Rahvar_2016_Am J Dermatopathol | 1 | 1 | 1 | 1 | 0 |  | 1 |  |  |
| Raja_2013_J Pak Med Assoc | 1 | 1 | 1 | 1 | 0 |  | 1 |  |  |
| Rashmi_2014_J Clin Diagn Res | 1 | 1 | 1 | 1 | 0 |  | 1 |  |  |
| Schussler_2018_Adv Genomics Genetics | 1 | 1 | 1 | 0 | 1 |  |  | 1 |  |
| Shieh_2006_Pediatrics | 3 | 3 | 3 | 2 | 1 | *no stratification of patients as ISH vs. JHF* | | | |
| Sugiura_2016_JEADV | 1 | 1 | 1 | 1 | 0 | *no stratification of patient as ISH vs. JHF* | | | |
| Soni_2016_Indian J Pediatr | 4 | 5 | 5 | 1 | 4 | 1 |  | 4 |  |
| Tanaka_2009_J Dermatol Sci | 1 | 1 | 1 | 1 | 0 |  | 1 |  |  |
| Temtamy_2015_Middle East J Med Genet | 1 | 3 | 3 | 1 | 2 | 1 |  | 2 |  |
| Tümer_2013_J Dermatol | 1 | 1 | 1 | 1 | 0 | *no stratification of patient as ISH vs. JHF* | | | |
| Vahidnezhad_2015_Clinm Exp Dermatol | 1 | 1 | 1 | 0 | 1 |  |  | 1 |  |
| Wang_2011_J Pediatr Hematol Oncol | 1 | 1 | 1 | 1 | 0 |  | 1 |  |  |
| Youseffian_2017_Acta Derm Venerol | 4 | 4 | 4 | 2 | 2 | *no stratification of patients as ISH vs. JHF* | | | |
| Youssefian_2018_BMC Med Genet | 4 | 4 | 4 | 2 | 2 | *no stratification of patients as ISH vs. JHF* | | | |
| **all previous studies total** | **74** | **100** | **72** | **36** | **36** | **14** | **13** | **19** | **6** |
| **present study** | **19** | **19** | **18** | **6** | **12** | *no stratification of patients as ISH vs. JHF* | | | |

^a^ considering partial overlap to Hanks et al. [2003]

^b^ considering complete overlap to Aggarwal et al. [2016]

^c^ considering complete overlap to Olczak-Kowalczyk et al. [2011]

references for Supplementary Table 2:

Aggarwal MLS, Chilakamarri V, Chennuri VS, Karra M. Identical Twins with Infantile Systemic Hyalinosis: Case study and review of literature. J Orthop case reports 6:69–71.

Sinani S Al, Murshedy F Al, Abdwani R. 2013. Infantile Systemic Hyalinosis: A Case Report with a Novel Mutation. Oman Med J 28:53–55.

Antaya RJ, Cajaiba MM, Madri J, Lopez MA, Ramirez MCM, Martignetti JA, Reyes-Múgica M. 2007. Juvenile hyaline fibromatosis and infantile systemic hyalinosis overlap associated with a novel mutation in capillary morphogenesis protein-2 gene. Am J Dermatopathol 29:99–103.

Casas-Alba D, Martínez-Monseny A, Pino-Ramírez RM, Alsina L, Castejón E, Navarro-Vilarrubí S, Pérez-Dueñas B, Serrano M, Palau F, García-Alix A. 2018. Hyaline fibromatosis syndrome: Clinical update and phenotype-genotype correlations. Hum Mutat. Epub ahead of print as doi:10.1002/humu.23638

Denadai R, Raposo-Amaral CE, Bertola D, Kim C, Alonso N, Hart T, Han S, Stelini RF, Buzzo CL, Raposo-Amaral CA, Hart PS. 2012. Identification of 2 novel ANTXR2 mutations in patients with hyaline fibromatosis syndrome and proposal of a modified grading system. Am J Med Genet A 158A:732–42.

Deuquet J, Lausch E, Guex N, Abrami L, Salvi S, Lakkaraju A, Ramirez MCM, Martignetti JA, Rokicki D, Bonafe L, Superti-Furga A, Goot FG van der. 2011. Hyaline Fibromatosis Syndrome inducing mutations in the ectodomain of anthrax toxin receptor 2 can be rescued by proteasome inhibitors. EMBO Mol Med 3:208–221.

Dowling O, Difeo A, Ramirez MC, Tukel T, Narla G, Bonafe L, Kayserili H, Yuksel-Apak M, Paller AS, Norton K, Teebi AS, Grum-Tokars V, et al. 2003. Mutations in capillary morphogenesis gene-2 result in the allelic disorders juvenile hyaline fibromatosis and infantile systemic hyalinosis. Am J Hum Genet 73:957–66.

El-Kamah GY, Fong K, El-Ruby M, Affifi HH, Clements SE, Lai-Cheong JE, Amr K, El-Darouti M, McGrath JA. 2010. Spectrum of mutations in the ANTXR2 (CMG2) gene in infantile systemic hyalinosis and juvenile hyaline fibromatosis. Br J Dermatol 163:213-5.

Fong K, Rama Devi AR, Lai-Cheong JE, Chirla D, Panda SK, Liu L, Tosi I, McGrath JA. 2012. Infantile systemic hyalinosis associated with a putative splice-site mutation in the ANTXR2 gene. Clin Exp Dermatol 37:635–638.

Haidar Z, Temanni R, Chouery E, Jithesh P, Liu W, Al-Ali R, Wang E, Marincola FM, Jalkh N, Haddad S, Haidar W, Chouchane L, et al. 2017. Diagnosis implications of the whole genome sequencing in a large Lebanese family with hyaline fibromatosis syndrome. BMC Genet 18:3.

Hakki SS, Ataoglu T, Avunduk MC, Erdemli E, Gunhan O, Rahman N. 2005. Periodontal treatment of two siblings with juvenile hyaline fibromatosis. J Clin Periodontol 32:1016–21.

Hanks S, Adams S, Douglas J, Arbour L, Atherton DJ, Balci S, Bode H, Campbell ME, Feingold M, Keser G, Kleijer W, Mancini G, et al. 2003. Mutations in the gene encoding capillary morphogenesis protein 2 cause juvenile hyaline fibromatosis and infantile systemic hyalinosis. Am J Hum Genet 73:791–800.

Hatamochi A, Sasaki T, Kawaguchi T, Suzuki H, Yamazaki S. 2007. A novel point mutation in the gene encoding capillary morphogenesis protein 2 in a Japanese patient with juvenile hyaline fibromatosis. Br J Dermatol 157:1037–9.

Huang Y-C, Xiao Y-Y, Zheng Y-H, Jang W, Yang Y-L, Zhu X-J. 2007. Infantile systemic hyalinosis: a case report and mutation analysis in a Chinese infant. Br J Dermatol 156:602–604.

Jaouad IC, Guaoua S, Hajjioui A, Sefiani A. 2014. Hyaline fibromatosis syndrome with mutation c.1074delT of the CMG2 gene: A case report. J Med Case Rep 8:291.

Koonuru MK, Venugopal SP. 2015. Infantile systemic hyalinosis in identical twins. Intractable Rare Dis Res 4:210–3.

Krasuska-Sławińska E, Polnik D, Rokicki D, Koeber B. 2015. Treatment of Massive Labial and Gingival Hypertrophy in a Patient With Infantile Systemic Hyalinosis—A Case Report. J Oral Maxillofac Surg 73:1962.e1-1962.e5.

Lee JY-Y, Tsai Y-M, Chao S-C, Tu Y-F. 2005. Capillary morphogenesis gene-2 mutation in infantile systemic hyalinosis: ultrastructural study and mutation analysis in a Taiwanese infant. Clin Exp Dermatol 30:176–9.

Lindvall LE, Kormeili T, Chen E, Ramirez MCM, Grum-Tokars V, Glucksman MJ, Martignetti JA, Zaragoza M V, Dyson SW. 2008. Infantile systemic hyalinosis: Case report and review of the literature. J Am Acad Dermatol 58:303–7.

Mallet S, Boye T, Hesse S, Fournier B, Guennoc B, Carsuzaa F. 2010. [Juvenile hyaline fibromatosis]. Ann Dermatol Venereol 137:364–8.

Mohamed S, Ahmed W, Al-Jurayyan N, Faqeih E, Al-Nemri A, Al-Ghamdi M. 2017. Infantile Systemic Hyalinosis Complicated with Right Atrial Thrombus and Pericardial Effusion in an Infant. Pediatr Neonatol 58:77–80.

Narayanan DL, Phadke SR. 2016. Infantile Systemic Hyalinosis with Mutation in ANTXR2. Indian J Pediatr 83:1356–1357.

Olczak-Kowalczyk D, Krasuska-Slawinska E, Rokicki D, Pronicki M. 2011. Case report: Infantile systemic hyalinosis: a dental perspective. Eur Arch Paediatr Dent 12:224–6.

Pena LDM, Jiang Y-H, Schoch K, Spillmann RC, Walley N, Stong N, Rapisardo Horn S, Sullivan JA, McConkie-Rosell A, Kansagra S, Smith EC, El-Dairi M, et al. 2018. Looking beyond the exome: a phenotype-first approach to molecular diagnostic resolution in rare and undiagnosed diseases. Genet Med 20:464–469.

Pirgon O, Atabek ME, Esen HH, Cangul H. 2007. Infantile systemic hyalinosis with early thyroid dysfunction. J Pediatr Endocrinol Metab 20:833–6.

Rahvar M, Teng J, Kim J. 2016. Systemic Hyalinosis With Heterozygous CMG2 Mutations: A Case Report and Review of Literature. Am J Dermatopathol 38:e60-3.

Raja K, Khan MA, Mubarak M, Abbas Z, Luck NH, Hassan SM. 2013. Three years old child with juvenile hyaline fibromatosis presenting with rectal bleeding. J Pak Med Assoc 63:396–8.

Rashmi M V., Geetha JP, Arava S, Niranjana Murthy B, Kodandaswamy CR. 2014. Juvenile hyaline fibromatosis: a rare case with recurrence. J Clin Diagn Res 8:161-2.

Schussler E, Linkner R V, Levitt J, Mehta L, Martignetti JA, Oishi K. 2018. Protein-losing enteropathy and joint contractures caused by a novel homozygous ANTXR2 mutation. Adv Genomics Genet 8:17–21.

Shieh JTC, Swidler P, Martignetti JA, Ramirez MCM, Balboni I, Kaplan J, Kennedy J, Abdul-Rahman O, Enns GM, Sandborg C, Slavotinek A, Hoyme HE. 2006. Systemic hyalinosis: a distinctive early childhood-onset disorder characterized by mutations in the anthrax toxin receptor 2 gene (ANTRX2). Pediatrics 118:e1485-92.

Soni JP, Puri RD, Jetha K, Bhavani GSL, Chaudhary M, Kohli S, Verma IC. 2016. Infantile Systemic Hyalinosis: Novel Founder Mutation in the Initiation Codon among “Malis (Farmers)” in Jodhpur. Indian J Pediatr 83:1341–1345.

Sugiura K, Ohno A, Kono M, Kitoh H, Itomi K, Akiyama M. 2016. Hyperpigmentation over the metacarpophalangeal joints and the malleoli in a case of hyaline fibromatosis syndrome with ANTXR2 mutations. J Eur Acad Dermatol Venereol 30:e44–e46.

Tanaka K, Ebihara T, Kusubata M, Adachi E, Arai M, Kawaguchi N, Utsunomiya J, Miki Y, Hiramoto M, Hattori S, Irie S. 2009. Abnormal collagen deposition in fibromas from patient with juvenile hyaline fibromatosis. J Dermatol Sci 55:197–200.

Temtamy SA, Aglan MS, Otaify GA, Abdel-Hamid M, Ismail S, Makrythanasis P, Hamamy H, Antonarakis SE. 2015. Exome sequencing in the diagnosis of an atypical phenotype of infantile hyalinosis. Middle East J Med Genet 4:18–23.

Tümer L, Kasapkara C, Fong K, Serdaroğlu A, McGrath JA. 2013. Hyaline fibromatosis syndrome resulting from a new homozygous missense mutation, p.Gly116Val, in ANTXR2. J Dermatol 40:677–8.

Vahidnezhad H, Ziaee V, Youssefian L, Li Q, Sotoudeh S, Uitto J. 2015. Infantile systemic hyalinosis in an Iranian family with a mutation in the *CMG2/ANTXR2* gene. Clin Exp Dermatol 40:636–639.

Wang Y-Y, Wen C-Q, Wei Z, Jin X. 2011. A novel splice site mutation in ANTXR2 (CMG2) gene results in systemic hyalinosis. J Pediatr Hematol Oncol 33:e355-7.

Youssefian L, Vahidnezhad H, Aghighi Y, Ziaee V, Zeinali S, Abiri M, Uitto J. 2017. Hyaline Fibromatosis Syndrome: A Novel Mutation and Recurrent Founder Mutation in the CMG2/ANTXR2 Gene. Acta Derm Venereol 97:108–109.

Youssefian L, Vahidnezhad H, Touati A, Ziaee V, Saeidian AH, Pajouhanfar S, Zeinali S, Uitto J. 2018. The genetic basis of hyaline fibromatosis syndrome in patients from a consanguineous background: a case series. BMC Med Genet 19:87.
