# Supplementary material for: Genetic, clinical and biochemical characterization of a large cohort of patients with hyaline fibromatosis syndrome
Source: Orphanet J Rare Dis. 2019 Aug 27;14:209. doi: 10.1186/s13023-019-1183-5 (PMC6712857; doi:10.1186/s13023-019-1183-5)
Supplement: Supplementary file 3 — Table S3. References for previous reports on ANTXR2 variants that were also identified by the present study (compare Table 2). (DOCX 16 kb) [file 13023_2019_1183_MOESM3_ESM.docx]

**Supplementary Table 3.** References for previous reports on *ANTXR2* variants that were also identified by the present study (compare Table 2).

| **variant** | **predicted consequence** | **previously reported by ^a^** |
| --- | --- | --- |
| c.134T>C | p.L45P | Hanks et al., 2003; Mohamed et al., 2017 |
| c.652T>C | p.C218R | Hanks et al., 2003 |
| c.1073delC | p.P358Lfs*51 | Lu et al., 2016 |
| c.1073dupC | p.A359Cfs*13 | Hanks et al., 2003; Dowling et al., 2003; Lee et al., 2005; Shieh et al., 2006; Deuquet et al., 2011; Denadai et al., 2012; Krasuska-Slawinska et al., 2015; Sigiura et al., 2016; Narayanan and Phadke, 2016; Rahvar et al., 2016; Pena et al., 2017; Youseffian et al., 2017; |
| c.1074delT | p.A359Hfs*50 | Hanks et al., 2003; Huang et al., 2007; Hatamochi et al., 2007; El-Kamah et al., 2010; Deuquet et al., 2011; Denadai et al., 2012; Jaoud et al., 2014; Vahidnezad et al., 2015; Youseffian et al., 2017 |
| c.1294C>T | p.R432* | Shieh et al., 2006; Sigiura et al., 2016; Rahvar et al., 2016 |
| c.1180-?_1428+?del | p.V394_490del | Denadi et al., 2012 |

^a^ for full bibliography see below

references for Supplementary Table 3:

Denadai R, Raposo-Amaral CE, Bertola D, Kim C, Alonso N, Hart T, Han S, Stelini RF, Buzzo CL, Raposo-Amaral CA, Hart PS. 2012. Identification of 2 novel ANTXR2 mutations in patients with hyaline fibromatosis syndrome and proposal of a modified grading system. Am J Med Genet A 158A:732–42.

Deuquet J, Lausch E, Guex N, Abrami L, Salvi S, Lakkaraju A, Ramirez MCM, Martignetti JA, Rokicki D, Bonafe L, Superti-Furga A, Goot FG van der. 2011. Hyaline Fibromatosis Syndrome inducing mutations in the ectodomain of anthrax toxin receptor 2 can be rescued by proteasome inhibitors. EMBO Mol Med 3:208–221.

Dowling O, Difeo A, Ramirez MC, Tukel T, Narla G, Bonafe L, Kayserili H, Yuksel-Apak M, Paller AS, Norton K, Teebi AS, Grum-Tokars V, et al. 2003. Mutations in capillary morphogenesis gene-2 result in the allelic disorders juvenile hyaline fibromatosis and infantile systemic hyalinosis. Am J Hum Genet 73:957–66.

El-Kamah GY, Fong K, El-Ruby M, Affifi HH, Clements SE, Lai-Cheong JE, Amr K, El-Darouti M, McGrath JA. 2010. Spectrum of mutations in the ANTXR2 (CMG2) gene in infantile systemic hyalinosis and juvenile hyaline fibromatosis. Br J Dermatol 163:213-5.

Hanks S, Adams S, Douglas J, Arbour L, Atherton DJ, Balci S, Bode H, Campbell ME, Feingold M, Keser G, Kleijer W, Mancini G, et al. 2003. Mutations in the gene encoding capillary morphogenesis protein 2 cause juvenile hyaline fibromatosis and infantile systemic hyalinosis. Am J Hum Genet 73:791–800.

Hatamochi A, Sasaki T, Kawaguchi T, Suzuki H, Yamazaki S. 2007. A novel point mutation in the gene encoding capillary morphogenesis protein 2 in a Japanese patient with juvenile hyaline fibromatosis. Br J Dermatol 157:1037–9.

Huang Y-C, Xiao Y-Y, Zheng Y-H, Jang W, Yang Y-L, Zhu X-J. 2007. Infantile systemic hyalinosis: a case report and mutation analysis in a Chinese infant. Br J Dermatol 156:602–604.

Jaouad IC, Guaoua S, Hajjioui A, Sefiani A. 2014. Hyaline fibromatosis syndrome with mutation c.1074delT of the CMG2 gene: A case report. J Med Case Rep 8:291.

Krasuska-Sławińska E, Polnik D, Rokicki D, Koeber B. 2015. Treatment of Massive Labial and Gingival Hypertrophy in a Patient With Infantile Systemic Hyalinosis—A Case Report. J Oral Maxillofac Surg 73:1962.e1-1962.e5.

Lee JY-Y, Tsai Y-M, Chao S-C, Tu Y-F. 2005. Capillary morphogenesis gene-2 mutation in infantile systemic hyalinosis: ultrastructural study and mutation analysis in a Taiwanese infant. Clin Exp Dermatol 30:176–9.

Mohamed S, Ahmed W, Al-Jurayyan N, Faqeih E, Al-Nemri A, Al-Ghamdi M. 2017. Infantile Systemic Hyalinosis Complicated with Right Atrial Thrombus and Pericardial Effusion in an Infant. Pediatr Neonatol 58:77–80.

Narayanan DL, Phadke SR. 2016. Infantile Systemic Hyalinosis with Mutation in ANTXR2. Indian J Pediatr 83:1356–1357.

Pena LDM, Jiang Y-H, Schoch K, Spillmann RC, Walley N, Stong N, Rapisardo Horn S, Sullivan JA, McConkie-Rosell A, Kansagra S, Smith EC, El-Dairi M, et al. 2018. Looking beyond the exome: a phenotype-first approach to molecular diagnostic resolution in rare and undiagnosed diseases. Genet Med 20:464–469.

Rahvar M, Teng J, Kim J. 2016. Systemic Hyalinosis With Heterozygous CMG2 Mutations: A Case Report and Review of Literature. Am J Dermatopathol 38:e60-3.

Shieh JTC, Swidler P, Martignetti JA, Ramirez MCM, Balboni I, Kaplan J, Kennedy J, Abdul-Rahman O, Enns GM, Sandborg C, Slavotinek A, Hoyme HE. 2006. Systemic hyalinosis: a distinctive early childhood-onset disorder characterized by mutations in the anthrax toxin receptor 2 gene (ANTRX2). Pediatrics 118:e1485-92.

Sugiura K, Ohno A, Kono M, Kitoh H, Itomi K, Akiyama M. 2016. Hyperpigmentation over the metacarpophalangeal joints and the malleoli in a case of hyaline fibromatosis syndrome with ANTXR2 mutations. J Eur Acad Dermatol Venereol 30:e44–e46.

Vahidnezhad H, Ziaee V, Youssefian L, Li Q, Sotoudeh S, Uitto J. 2015. Infantile systemic hyalinosis in an Iranian family with a mutation in the *CMG2/ANTXR2* gene. Clin Exp Dermatol 40:636–639.

Youssefian L, Vahidnezhad H, Aghighi Y, Ziaee V, Zeinali S, Abiri M, Uitto J. 2017. Hyaline Fibromatosis Syndrome: A Novel Mutation and Recurrent Founder Mutation in the CMG2/ANTXR2 Gene. Acta Derm Venereol 97:108–109.
